# Preceptors’ perception of their role as educators and professionals in a health system

**DOI:** 10.1186/s12909-019-1642-7

**Published:** 2019-06-13

**Authors:** Letícia Cabrini Girotto, Sylvia Claassen Enns, Marilda Siriani de Oliveira, Fernanda Brenneisen Mayer, Bruno Perotta, Itamar Souza Santos, Patricia Tempski

**Affiliations:** 1 Departamento Regional de Saúde de Marília, Rua Quinze de Novembro, 1151, Alto Cafezal, Marília, SP 17504000 Brazil; 2 Instituto de Ensino e Pesquisa Hospital do Coração, Rua Desembargador Eliseu Guilherme, 390, Paraíso, São Paulo, SP 04004030 Brazil; 3 Centro de Desenvolvimento de Educação Médica, Faculdade de Medicina da Universidade de São Paulo, Av. Dr. Arnaldo, 455 - Sala 2345, São Paulo, SP 01246-903 Brazil; 4 Pontifícia Universidade Católica do Paraná, Rua Imaculada Conceição 1155, bloco 6, Prado Velho, Curitiba, PR 80215901 Brazil; 5 Faculdade Evangélica do Paraná, Rua Padre Anchieta, 2770, Bigorrilho, Curitiba, PR 80730000 Brazil; 6 Faculdade de Medicina Universidade de São Paulo, Av. Dr. Arnaldo, 455 - Sala 2345 –, São Paulo, SP 01246-903 Brazil

**Keywords:** Preceptorship, Health human resource training, Competence, professional, Teaching care integration services, Unified health system

## Abstract

**Background:**

Preceptorship fulfills the requirements of International Guidelines regarding the training of health care professionals as a method of teaching in clinical settings, during the daily work routine. This study aims to analyze the preceptors’ perceptions about preceptorship and their role as educators.

**Methods:**

Data were collected via a questionnaire with 35 five-point Likert-type scale statements and analyzed using quantitative and qualitative approaches. The qualitative analysis consisted of two open-ended questions: (1) What is Preceptorship? And (2) What is your perception of the preceptor’s role as an educator?

**Results:**

Out of 619 invited Brazilian preceptors from different health care professions, 327 (52.8%) participated in the study. Among them, 80.7% were females, 35.2% were nurses and 8.9% were physicians. Factor analysis revealed five factors: Pedagogical Competence (F1), Support and educational resources (F2), Educational program planning (F3), Teaching-service integration (F4), and Student presence in the clinical setting (F5). About F1, F3, and F5, professionals from the northeast region had a more positive perception than professionals from the southeast. The item analysis revealed that preceptors learn from the students and consider the service network co-responsible for their training. However, they agreed that only a small part of the health care team participates in the program. Participants described preceptorship as an educational task in a clinical setting, in which active learning methods are used for the training of health care professionals. Preceptorship was considered a bridge between the Unified Health System and the Academic Practice. They envisioned their educator role as a model, tutor, leader, supervisor, and mentor.

**Conclusion:**

Preceptors expressed a critical view about the nature of preceptorship and their role as educators, recognizing its challenges as well as its potential in clinical settings.

## Background

Preceptorship may be defined as a simultaneous teaching-learning method used by the practice professions of nursing, medicine, pharmacy and dentistry in teaching students in clinical settings, focusing on their clinical and ethical development [1–3]. The preceptor is a professional with a generalist or specialist training, whose function is direct follow-up and orientation regarding the practical activities carried out by undergraduate and graduate students, while developing their assistance function. It is a close teaching-learning relationship, in which the preceptor acts as a model for the professional in training [4, 5]. In this context, preceptorship is essential to improve the quality of training and, consequently, health care.

Preceptorship was implemented in Brazil in 2001. It was established that the training and development of health care professionals should occur at different levels of care and in various settings of the Unified Health System (SUS), mainly in primary care [6]. SUS is a national public health care system funded by the government and offered to all Brazilian citizens, covering all levels of care [7]. Its mission also includes professional training in clinical settings through the preceptorship method, according to the federal curriculum guidelines for graduation [8]. Preceptors in Brazil work 1:1 with a student or in little groups with six to eight students.

The preceptorship occurs in clinical settings in the health care system, allowing students to experience health care, to interact with the professional team, and to be exposed to the communities’ reality [9], establishing a connection between what is learned from medical schools and society’s health care needs [10–13]. However, it requires the reorganization of operations and staff at the clinical site to include preceptors and students in their routine procedures [4, 14].

The preceptor plays an important role by guiding and role modelling their knowledge, skills and practice to increase confidence and enhance students’ practice, giving them the opportunity to be moulded through positive engagement into an autonomous, decision making practitioner [5]. This form of learning in practice, mediated by a preceptor, is conducive to the development of a critical awareness by the student about reality. It is expected that professional training in the twenty-first century leads students for applying a critical view to their work and, when necessary, capable of taking measures for the transformation of reality [15]. Thus, training in clinical settings can promote social responsibility in students who participate in preceptorship programs.

Since preceptors are at the center of this educational process, it is crucial to assess their perception of their educational practices in the health care system and their roles when training future health care professionals.

## Methods

### Study design

This cross-sectional study, with both a quantitative and qualitative approach, was approved by The Human Research Ethics Committee of the University of São Paulo. The participants were all volunteers and did not receive any remuneration or advantage. They also signed a written informed consent before data collection, which occurred between May and June of 2014.

### Participants

Participants came from a Postgraduate Program of Health Education for Preceptors offered by the Brazilian Health Ministry in partnership with the Teaching and Research Institute of the Hospital Sirio-Libanês. This program aims to develop professionals in health education for their role as preceptors in clinical settings in the Brazilian Unified Health System (SUS). Six hundred nineteen graduated preceptors from different health professions, like nursing, medicine, physiotherapy, social assistance, psychology, dentistry, pharmacy and others were invited to participate. They were employed in the Brazilian Unified Health System (SUS) located in 18 cities around the country.

### Instruments

Volunteers answered a sociodemographic questionnaire including variables related to gender, age, field of study, work-related experience, and working field (provider, management and/or education) and a questionnaire, developed by the researcher group. The elaboration of this instrument was based on an extensive review of the literature and consensus of three specialists in health education and preceptorship, who reviewed the material individually and together for validation of the construct. The questionnaire includes 35 statements scored with a five-point Likert scale: 1-totally disagree (TD), 2-disagree (D), 3-indifferent (I), 4-agree (A) to 5-totally agree (TA). Affirmations that represented negative aspects regarding the preceptorship had their values ​​inverted. The following issues were reversed: 1, 2, 4, 16, 20, 28 and 31. For example, in item 1 (The presence of the student in the work environment overloads my activities), 55.6% of the preceptors disagreed with the statement, which is presented in Table 1 as a positive view. It also included two open-ended questions: (1) What is Preceptorship? And (2) What is your perception of the preceptor’s role as an educator?

**Table 1:** Preceptor’s positive and negative perception in item analysis

Statements	Positive Perception (%)	Negative Perception (%)
1. The presence of the student in the work environment overloads my activities.	55.6	44.4
2. The presence of the student displeases the users.	67.5	32.5
3. The quality of my work improves with the students’ presence.	76.9	23.1
4. I have no autonomy to develop educational plans.	56.2	43.8
5. The service network is co-responsible for the health care students’ training.	90.7	9.3
6. My preceptorship activities follow the National Curriculum Guidelines.	77.8	22.2
7. I have the resources needed to develop my educational activities.	53.3	46.7
8. My preceptorship activities integrate the student in the health care team.	88.5	11.5
9. I had pedagogical training to develop the preceptorship.	55.4	44.6
10. I have my management’s support to develop the preceptorship.	73.8	26.2
11. I am able to develop educational activities.	90.7	9.3
12. The whole health care team participates in the students’ training.	44.9	55.1
13. I take part in the discussion forums of the Teaching-service integration.	63.6	36.4
14. My work activities were reorganized because of the students’ presence.	55.6	44.4
15. I know the curriculum of the course in which I am a preceptor.	64.5	35.5
16. The presence of the student compromises patient safety.	80.2	19.8
17. My preceptorship activity is recognized by University professionals.	57.8	42.2
18. My practice allows me to articulate the biological, social, and cultural aspects of the health-disease process.	86.1	13.9
19. I identify the population’s health care needs to establish educational goals.	85.1	14.9
20. My educational goals do not take the population’s health care needs into account.	82.4	17.6
21. I use database to keep updated.	89.5	10.5
22. My educational goals take attitudes, skills, and knowledge into account.	96.0	4.0
23. I know my students and take their previous knowledge into account.	79.4	20.6
24. I adopt the theoretical and practical correlation in the preceptorship.	94.4	5.6
25. I am aware of my own learning needs.	95.7	4.3
26. I always evaluate my student.	85.4	14.6
27. I evaluate my student at the end of the process.	72.0	28.0
28. The students’ evaluation is not my responsibility.	85.3	14.7
29. I learn from my students.	97.8	2.2
30. I develop research activities with the students.	74.4	25.6
31. The presence of the student generates conflicts in the team.	60.4	39.6
32. My workspace is adequate for the preceptorship.	46.0	54.0
33. I am paid to be a preceptor.	38.0	62.0
34. I am interested in pursuing a teaching career.	87.0	13.0
35. My activity as a preceptor improves my quality of life.	87.0	13.0

*Note.* Positive perception = TA and A; Negative perception = I, D and TD

### Data analysis

Descriptive statistics and Chi-square tests were applied using R version 3.1.1. The adopted significance level was .05. Data reliability was verified by the internal consistency of the instruments through Cronbach’s alpha coefficient. Values greater than or equal to 0.7 are considered satisfactory for study groups [16, 17]. Item analysis was conducted grouping the responses in two categories: positive (A + TA) and negative (TD + D + I) perception of the preceptorship. The quantitative analysis was complemented by a factor analysis using principal components and varimax rotation.

The qualitative analysis of the two open-ended questions followed traditional content analysis methods, that is preparation of the material (typing of the answers of the open questions and organization of the data), free reading, highlighting subjects by relevance and/or repetition, categorization of the emerging categories and derived issues, discussion with the research group, and a descriptive presentation of the results using quotes from the participants answers [18, 19].

## Results

**Fig. 1:**
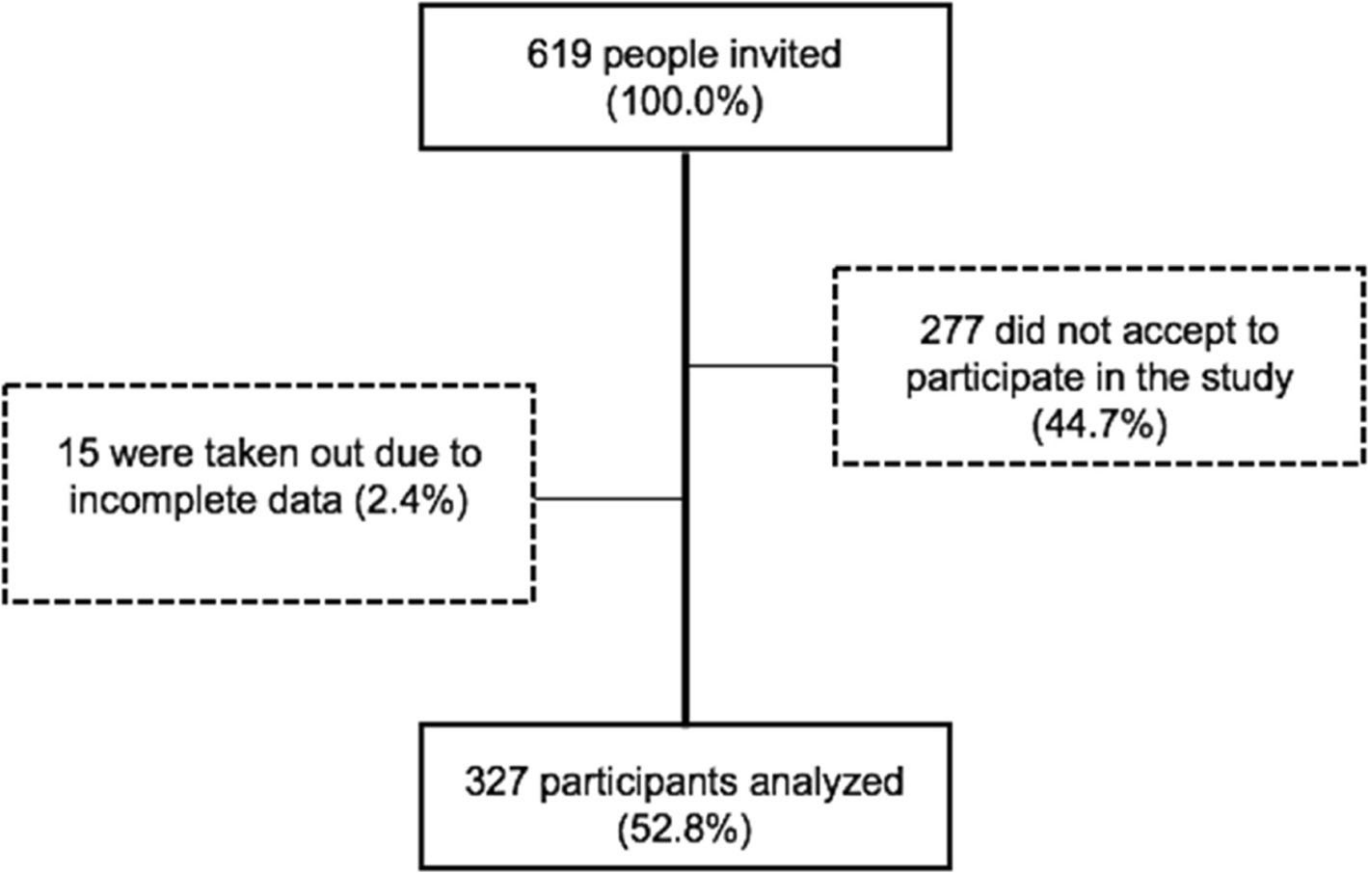
Study participants and losses flowchart

From 619 Brazilian preceptors, 327 (52.8%) participated in the study (Fig. 1). Among them 264 (80.7%) were females, 58 (17.7%) males and 5 (1.53%) did not indicate their gender. The sample included 115 (35.17%) nurses, 29 (8.9%) physicians, 26 (7.9%) physiotherapists, 22 (6.7%) social workers, 21 (6.4%) psychologists, 16 (4.9%) pharmacists, 14 (4.2%) dentists, and 42 (12.8%) professionals that were not from health professions, but from administrative areas. Among them, 21 (6.42%) had finished residency programs, 49 (15%) had master’s degree, and 11 (3.36%) were Ph.D. The length of experience in health care training varied between 0 to 38 years (median = 3). All participants worked in the Brazilian Health System (SUS) in three main areas: providers (221), health care management (93), and health care education (148).

Cronbach’s alpha coefficient was 0.84, suggesting good reliability of the questionnaire (data not shown). Table 1 details the positive and negative preceptors’ perception. While they were fully engaged in their teaching activities, preceptors had somewhat mixed feelings: on one hand, they feel overwhelmed and think that the presence of students is a risk to the patients. At the same time, they understand that preceptorship improves the quality of care, they feel interested and are prepared for the teaching activities, albeit underpaid for it. Some other statements showed a balance between positive and negative perceptions. Preceptors seem to be uncertain if they have the necessary resources (items 7 and 9), adequate workplace (item 32), or effective support from the other professionals (item 12).

The factor analysis, using principal components and varimax rotation, delimited five factors: F1-Pedagogical competence, F2-Support and educational resources, F3-Educational program planning, F4-Teaching-service integration, and F5-Student presence in the clinical setting. Professionals from the northeast region of the country had a more positive perception while professionals from the southeast had a more negative one regarding F1, F3, and F5 (Table 2). Pharmacists and professionals working as providers had a more negative perception about F1, F2, and F3 (Tables 3 and 4).

**Table 2:** Factorial analysis comparing country regions

Factor	Mean Score	Region					p*
		Northeast (SD)	North (SD)	Midwest (SD)	Southeast (SD)	South (SD)	
1 – Pedagogical Competence	35.0	44.6 (0.9)	44.5 (0.8)	43.1 (1.0)	42.1 (1.0)	42.8 (0.9)	< 0.05
2 – Support and educational resources	21.0	20.9 (1.4)	20.0 (1.4)	20.0 (1.4)	19.4 (1.4)	20.0 (1.3)	0.198
3 – Educational program planning	24.5	28.1 (1.2)	27.9 (1.2)	26.9 (1.3)	24.6 (1.4)	26.8 (1.2)	< 0.05
4 – Teaching-service integration	10.5	12.8 (1.1)	12.8 (1.0)	12.6 (1.0)	12.5 (1.0)	12.7 (1.0)	0.872
5 – Student presence in a clinical setting	31.5	35.3 (1.3)	33.4 (1.4)	33.8 (1.4)	31.7 (1.4)	33.3 (1.4)	< 0.05

*Note*. *ANOVA test

**Table 3:** Factorial analysis comparing preceptors’ professional fields

Factor	Mean Score	Nurs (SD)	Phy (SD)	Phys (SD)	Soc (SD)	Psyc. (SD)	Pharm (SD)	Dent (SD)	Other (SD)	p*
1 – Pedagogical Competence	35.0	43.3 (1.0)	42.9 (1.0)	44.4 (0.8)	46.3 (0.7)	43.5 (0.9)	41.9 (1.1)	44.1 (0.9)	43.1 (0.9)	< 0.05
2 – Support and educational resources	21.0	19.9 (1.4)	18.4 (1.5)	20.6 (1.4)	21.0 (1.4)	21.4 (1.3)	16.6 (1.4)	22.8 (1.2)	20.6 (1.4)	< 0.05
3 – Educational program planning	24.5	27.0 (1.3)	27.4 (1.2)	28.0 (1.2)	27.3 (1.1)	28.0 (1.1)	24.5 (1.5)	28.8 (1.1)	25.9 (1.2)	< 0.05
4 – Teaching-service integration	10.5	13.0 (1.0)	12.5 (1.1)	12,7 (0.9)	13.2 (1.0)	12.6 (0.9)	12.4 (1.2)	13.3 (0,8)	12.0 (1.1)	0.062
5 – Student presence in a clinical setting	31.5	34.1 (1.3)	33.6 (1.5)	34.3 (1.4)	34.1 (1.5)	33.5 (1.4)	32.6 (1.5)	34.2 (1.4)	33.1 (1.3)	0.786

*Note.* *ANOVA test. *Nur* Nurses, *Phy* Physicians, *Phys* Physiotherapists, *Soc* Social workers, *Psy* Psychologists, *Pharm* Pharmacists, *Dent* Dentists

**Table 4:** Factorial analysis comparing the professional area of preceptors

Factor	Mean Score	Providers (SD)	Management (SD)	Education (SD)	Two or more fields (SD)	p*
1 – Pedagogical Competence	35.0	42.6 (1.0)	44.0 (0.9)	43.2 (1.0)	44.6 (0.9)	< 0.05
2 – Support and educational resources	21.0	18.9 (1.4)	21.2 (1.3)	22.5 (1.2)	20.3 (1.4)	< 0.05
3 – Educational program planning	24.5	26.1 (1.3)	27.2 (1.7)	27.5 (1.2)	28.2 (1.2)	< 0.05
4 – Teaching-service integration	10.5	12.6 (1.0)	12.9 (1.0)	12.2 (1.2)	13.0 (0.9)	0.095
5 – Student presence in a clinical setting	31.5	33.4 (1.4)	32.7 (1.4)	34.3 (1.3)	34.3 (1.4)	0.091

*Note.* *ANOVA test

Answers to the first open-ended question - What is Preceptorship? - were organized in four categories: (1) Integration between teaching and health care services, (2) Teaching in clinical settings, (3) Active Methods, and (4) Reality changing (Table 5).

**Table 5:** Categories and issues for the meaning of the Preceptorship

Category	Issues	Comments
Integration of teaching and health care services	Co-responsibility	“The teaching and learning process integrates the student to the health care team, who is co-responsible for their professional development.” (Psychologist)
Teaching in clinical settings	Mediation	“The activity performed by the professional that is working in this field is to welcome, follow, stimulate and evaluate the students during their learning process, giving them what they need for their training.” (Nutritionist)
	Autonomy development	“Knowledge mediation process supported resulting in subject’s autonomy development.” (Nurse)
Active Methods	Practical learning	“It consists of the development of an educational environment, according to the National Curriculum Guidelines and the population’s health care needs.” (Social Worker)
	Educational Planning	“Activities which include the development of an educational plan during the training process: learning methods, evaluation, and feedback.” (Nurse)
Reality Changing	Critical Awareness	“It is to help the student’s training in a clinical setting, looking for the development of their reflection about concepts and their applications to the reality.” (Pharmacist)
	Knowledge Building	“The relationship between health care professionals and students, contributing to knowledge building, which benefits both of them, as well the patients.” (Physiotherapist)

Researchers noticed that study participants attributed to preceptorship the function of a mediator between the learning and teaching processes, in which active methods are used in clinical settings to train professionals to address the population’s health care needs. In this sense, the preceptors see the preceptorship as a bridge between teaching and the health care system. When students are in a clinical setting, they interact with the health care team and become responsible for their learning, participate in their educational planning, and contribute to the effectiveness of the curriculum from the educational institutions.

Preceptors believe that the practical learning process promotes students’ autonomy as well as the development of their critical awareness, allowing them to understand the communities’ needs and change the health care reality. This belief could be observed in the following sentence:

“*It is to help train the student in a clinical setting, looking for the development of their reflection about the concepts and their applications to reality.”* (Pharmacist)

For the second question - What is your perception of the preceptor’s role as an educator? - the emerged categories were (1) Educator, (2) Integrator of theory and practice, and (3) Commitment (Table 6).

**Table 6:** Categories and issues for the preceptors’ perception of their role as an educator

Category	Issues	Comments
Educator	Role Model	“To have a positive influence in the development of future professionals, giving them the opportunity to be a part of a participative, ethical and humanizing process.” (Social Worker)
	Tutor	“Leader, mentor, supervisor and tutor role.” (Nurse)
Integrator of theory and practice	Integration	“The preceptor helps the students to identify the population’s health needs, in the training process, allowing them to give their learning a better direction.” (Pharmacist)
Commitment	Reflection and Action	“Gets involved in the development of pedagogical activities to raise students’ critical awareness, never forgetting their roles as reality changers.” (Physician)

Preceptors see their roles as educators and understand their responsibility for providing better training beyond academics. The preceptors perceive themselves as an integral part of the health care professionals’ ethical and awareness development. They realize as well their influence as role models in the teaching and learning process. The results also showed that preceptors recognize the intrinsic value of their social role to preceptorship, as exemplified in the following phrase:

*“Getting involved in the development of pedagogical activities to raise students’ critical awareness, never forgetting their roles as reality-changers.”* (Physician)

## Discussion

This study addresses the need to understand preceptors’ perception of the preceptorship and to give voice to these professionals who work with both health care and education. All of them perceive their roles as educators and are aware of the potentialities of the health care training process in a clinical setting. This awareness was not associated with preceptor’s undergraduate degree, their main professional capacity, or their experience in the training of health care professionals.

In this study, the majority of preceptors worked as health care providers and confirmed that the primary objective of preceptorship is the training in clinical settings, as conceptualized in the literature [9]. Also, many of the participants work in two or more capacities such as management, education, and as health care provider.

The results re-emphasize that the preceptor is a professional who works in the health care system with general or specialized expertise, enhancing students’ practical activities during their undergraduate and post-graduate education, while performing their own role as health care professionals [4, 20, 21]. Preceptors are considered facilitators in the learning process who can integrate theory and practice [20, 22, 23].

One-on-one teaching helps further develop students’ skills and attitudes, emphasizing the inseparability between theory and practice, given that both are essential to critical thinking, leading to a holistic health care view [24]. This close relationship between students and preceptors transforms the latter into role models, a professional who inspires and promotes the development of others [25–27]. The qualitative data of this study showed that the main skills of the good preceptor, listed by the preceptors, were knowledge, experience, being accessible and having good communication.

Preceptorship is a process of adult learning: thus, it is necessary that the preceptor knows and applies andragogy principles [28]. Therefore, the students’ previous knowledge, their culture, and life experience must be considered during the training to develop their autonomy and their ability to critical thinking [20, 29]. This ability to reflect about the practice and reality is essential to the health care learning process. It prepares them for constant evaluation and changes in their future practice, leading them to search for better ways and tools to perform their work, making them better professionals [25, 30]. Our results showed that preceptors recognize the power of preceptorship to transform reality, as it provides students with autonomy and develops their critical vision. They perceive themselves as a professional model that favors the development of technical, emotional and moral competence, training students with commitment to the transformation of reality in which they are inserted and promotion of the quality of life of the population.

Concerning the partnership between educational institutions and the health care system, preceptors distinguish themselves as a link between the two. As such, this partnership entails a new way of training health care professionals in new educational settings where they can apply their knowledge. The study participants realize that preceptorship is a valuable way to provide this training.

The preceptorship is especially essential at this historical moment, when the national and international guidelines value learning in clinical settings, especially in primary health care [15, 31–33]. The analysis of the preceptors’ perceptions leads to the conclusion that, for many of them, their social role is intrinsic to the preceptorship concept. They perceive preceptorship as a good opportunity for a bidirectional development, helping the development of the future professional while contributing to their development. Many of them recognized improvements to their health care practice as a result of the students’ presence, questions, and suggestions.

On the other hand, the difficulties cannot be overlooked. The preceptors have often not received any training to act as educators and do not have extra time in their already overloaded schedule for teaching activities. The lack of infrastructure in the health care system and support from the health care team were also mentioned as difficulties faced by preceptors.

The strength of this study is the original research design, quantitative and qualitative approach and the national scope, having the representativeness of the five regions of Brazil, bringing new insights about preceptorship potentialities. On the other hand, it has the limitation that it is a cross-sectional analysis of a particular group of preceptors, so it may not represent the perception of every preceptor in Brazil.

## Conclusions

The preceptors in this study had a positive perception about preceptorship, with a clear vision of their role as educators, role models, tutors, advisors, supervisors, and mentors of other professionals in a clinical setting. Preceptors recognized that preceptorship fulfills the requirements of Brazilian’s present guidelines for professional training.

The most challenging difficulties stated by the participants were the lack of infrastructure in the health care system, of support from the health care team, and of payment for preceptorship. The data analysis leads to the conclusion that resources and the reality of preceptorship are different around the country. It shows that much must be done for the development and improvement of better learning in the health care system.

## Data Availability

The datasets used and/or analysed during the current study are available from the corresponding author on reasonable request.

## Abbreviation

SUS: Sistema Único de Saúde (Unified Health System)
